# Cardiorespiratory Aerobic Fitness and Repeated Sprint Ability in Elite Ice Hockey Players

**DOI:** 10.3390/sports14070287

**Published:** 2026-07-07

**Authors:** Jan Malecha, Libor Staněk, Vladimir Tuka, Martin Sedlář, Jiří Suchý, Agáta Jeníšová, Aleš Linhart

**Affiliations:** 1 ProCorde Inc., 430 01 Chomutov, Czech Republic; malecha@email.cz; 2 2nd Department of Internal Cardiovascular Medicine, General University Hospital in Prague, First Faculty of Medicine, Charles University, 128 08 Prague, Czech Republic; kardiologie.chomutov@email.cz; 3 Institute of Sport Medicine, General University Hospital in Prague, First Faculty of Medicine, Charles University, 128 08 Prague, Czech Republic; vladimir.tuka@lf1.cuni.cz; 4 1st Department of Surgery, General University Hospital in Prague, First Faculty of Medicine, Charles University, 128 08 Prague, Czech Republic; martin.sedlar@lf1.cuni.cz; 5 Department of Physical Education, Faculty of Education, Charles University, 116 39 Prague, Czech Republic; jiri.suchy@pedf.cuni.cz; 6 2nd Department of Medicine—Department of Cardiovascular Medicine, General University Hospital in Prague, First Faculty of Medicine, Charles University, 128 08 Prague, Czech Republic; agata.beznoskova@lf1.cuni.cz

**Keywords:** ice hockey, cardiovascular physiology, cardiopulmonary exercise stress testing

## Abstract

Ice hockey represents a sport with predominantly anaerobic efforts best reflected by repeated sprint ability (RSA) testing (5 × 5 s with 10 s recovery). A controversy persists about the usefulness of VO_2_max laboratory testing for the assessment of ice hockey players. The purpose of this study was to evaluate the relationship between laboratory-measured VO_2max_ and RSA simulated on a supine ergometer and tested on ice. Elite male hockey players (*n* = 64) were tested in the laboratory (VO_2max_ and RSA). RSA testing was performed by a modified Wingate test (5 × 5 s sprints with 10 s recovery). In 28 athletes RSA was assessed during on-ice testing (five maximal skating sprints between the goal and the blue line). The decrease in performance was assessed by fatigue indices. In the laboratory setting, VO_2_max correlated significantly with maximum workloads of the second, third, fourth and fifth bouts with increasing correlation strength (r = 0.26, *p* = 0.02; r = 0.48, *p* < 0.001; r = 0.57, *p* < 0.001; and r = 0.60, *p* < 0.001) and with fatigue indices—the percentage workload decrement index (r = 0.44, *p* < 0.001) and percentage maximum average workload decrement (%) (r = 0.38, *p* = 0.002). In addition, VO_2_max correlated with lactate levels after 10 min of recovery (r = 0.31, *p* = 0.01). There was no correlation between VO_2_max and on-ice testing results. Moreover, the results of RSA measured in the laboratory and on ice did not show any correlation. The lack of relationship between laboratory and on-ice testing further challenges the usefulness of bicycle ergometry laboratory testing in ice hockey.

## 1. Introduction

Ice hockey represents one of the most physically demanding sports. The high-intensity skating during individual shifts typically represents an anaerobic effort requiring muscle strength, power, and anaerobic endurance. However, endurance fitness plays a major role as the typical player performs from 15 to 20 min in repeated shifts of 30 to 80 s with a brief 3 to 5 min recovery time between them. The length of the game and the need to quickly recover between shifts may heavily depend on the quality of the aerobic system [1]. Aerobic metabolism has been reported to contribute significantly to recovery, particularly during the first 30–120 s following exercise [2]. This time interval closely corresponds to the time players typically spend on the bench between shifts.

Although several on-ice tests have been proposed to evaluate the fitness of ice hockey players [3], many professional hockey teams require laboratory testing and measurement of maximal oxygen uptake (VO_2_max) for the fitness assessment of players (e.g., recommended by the Czech Ice Hockey Association for the U17 and U20 categories).

There is an ongoing debate on how useful VO_2_max assessment may be for anaerobic activities such as ice hockey. It has been proposed that the oxidative capacity of the muscle is the primary component of the aerobic energy system affecting recovery after repeated sprint activities [4]. Several smaller studies reported only a weak association between VO_2_max measurements and repeated sprint ability (RSA). This was observed in male field hockey and soccer players (*n* = 40, national level) and female ice hockey players (*n* = 11, college level) [5,6]. In contrast, other authors have reported that aerobic capacity may significantly influence RSA [2,7,8]. A positive relationship between VO_2_max and RSA was recently reported in ice hockey players (*n* = 45, college level) as well when on-ice repeated shift results were compared with VO_2_max measured on a skating treadmill [9]. Surprisingly, VO_2_max was shown to predict amateur ice hockey players’ (*n* = 29, college level) scoring chances [10]. There could be various factors explaining the discrepancy in the results (e.g., sample size, RSA testing methods, participant motivation) [9]. In ice hockey, performance relies heavily on skating technique, which introduces variability in test outcomes. Although laboratory testing may reduce this variability, the ability of off-ice fitness tests to predict ice hockey performance has been questioned [11]. Another factor is the game’s frequent changes in direction, which increase energy demands [12]. Finally, VO_2_max testing on a treadmill or supine cycle ergometer may not be appropriate for ice hockey players [13]. Because VO_2_max alone does not fully capture the intermittent, high-intensity nature of ice hockey performance, assessment methods focusing on anaerobic power and repeated sprint ability have been increasingly emphasized.

One of the most widely used laboratory methods for assessing anaerobic performance is the Wingate Anaerobic Test (WT) [14]. The WT consists of 30 s maximal (“all-out”) cycling against a constant braking force. Although longer protocols have been proposed [15], the standard 30 s test remains the most commonly used. However, the continuous nature of the WT differs substantially from the intermittent high-intensity activity pattern of ice hockey, which is characterized by repeated short-duration skating efforts interspersed with brief recovery periods [1].

To better reflect these sport-specific demands, repeated sprint ability (RSA) protocols have been developed, involving repeated maximal efforts of short duration with limited recovery. Previous research has demonstrated that RSA testing provides acceptable reliability and validity for the assessment of repeated high-intensity performance [16,17,18]. Nevertheless, on-ice testing incorporates skating-specific technical demands that may influence performance outcomes, whereas laboratory-based assessments provide greater control over testing conditions and minimize the influence of skating technique. Recent reviews have highlighted that on-ice and off-ice testing offer complementary information regarding the physical performance capacities of ice hockey players [3].

This study aimed at addressing three questions that have affinity with the physiological demands of ice hockey:

(1) Whether laboratory measurements of VO_2_max are related to RSA as assessed by laboratory and on-ice testing;

(2) Whether the laboratory and on-ice methods of repeated sprint ability testing are related and comparable;

(3) Whether VO_2_max relates to recovery after sprints as assessed by lactate level measurements subsequent to testing.

## 2. Materials and Methods

### 2.1. Design of the Research

Two groups were recruited for this study: elite adult professional ice hockey players (*n* = 45) and elite junior players (*n* = 19), all competing at the highest national level. The study aimed to characterize the general performance profile of elite ice hockey players; therefore, data from all participants were pooled for the primary analyses, with additional subgroup analyses performed separately for senior and junior players to enable more detailed comparison.

Testing was conducted during the final phase of structured pre-season (summer) training preceding the competitive league season in elite ice hockey players competing at the highest national level. The average training time during the camp was approximately 30 h per seven-day micro cycle. All participants had one day of rest before being tested and were told to refrain from heavy exercise for 24 h prior to their testing session.

To exclude any significant cardiovascular pathology, all subjects underwent a clinical examination, ECG assessment and a standard echocardiographic examination. These were performed according to a 2005 recommendation by the American Society of Echocardiography and European Association of Echocardiography using Vivid 7 equipment (General Electric Ultrasound, Horten, Norway). Body mass and composition were evaluated by InBody720 (InBody Co. Ltd., Seoul, Republic of Korea).

Cardiopulmonary exercise stress testing was performed on a Lode Excalibur supine ergometer (Lode, Groningen, The Netherlands) using the Innocor P10 system (Innovision, Glamsbjerg, Denmark). The system was calibrated before each examination. The test was conducted in a dedicated laboratory under controlled environmental conditions, with the ambient temperature maintained at 23 °C and relative humidity set at 45%. The lactate concentration was measured from a finger prick using a Lactate Pro device (Arkray Europe, Amstelveen, The Netherlands).

The laboratory RSA testing was performed using a modification of the WT and bicycle sprint tests [18]. All participants were instructed to complete their typical individual off-ice pre-training warm-up (duration: 10 min). Before the test the position of the saddle was adjusted in line with the subject’s wishes, and the subject’s feet were strapped to the pedals [19]. The testing protocol was initiated with a 5 min warm-up using a low-intensity load of 1 W/kg. Then a series of five simulated sprints were performed using the modified WT set to a torque factor calculated as 0.9 * subject’s body weight. Five repeated sprints of 5 s were interspersed with 10 s of recovery with unloaded pedalling. The test was followed by a 10 min recovery period with a low-intensity (0.5 W/kg) workload on the bicycle ergometer [18], during which lactate sampling was performed at the 3rd, 5th, and 10th min. Lactate samples taken after RSA tests on an ergometer reflect the kinetics of active recovery during low-intensity exercise, not passive recovery after exercise. After completion of the 10 min low-intensity workload (0.5 W·kg^−1^) and a 1 min technical transition, a standardized cardiopulmonary exercise test was initiated, starting at 1 W/kg for 4 min, then 1.5 W/kg for 4 min, and subsequently increasing by 0.25 W/kg every 30 s until maximal exhaustion. On completion of the cardiopulmonary exercise test a final blood lactate sample was collected 3 min post to assess the maximum accumulated blood lactate concentration.

All participants in the study had the possibility to perform the one-ice testing. Of the 32 participants who provided informed consent, four netminders were excluded from the analysis due to limitations imposed by their protective equipment.

The test was performed within a week after the laboratory testing (minimum interval of 2 days, maximum of 9 days). All participants were instructed to have their ice skates sharpened and to complete their typical pre-game warm-up (duration: 10 min). The on-ice test comprised five maximal-intensity skating sprints performed between the goal line and the far blue line (approximately 34 m), conducted in full hockey equipment (including the stick).

Each sprint effort lasted approximately 5 s beginning from a stationary 2-point position, with 10 s of passive active recovery between repetitions returning to the starting position. The time was measured using laser timer equipment. After the test’s completion, the lactate concentration was measured at the 3rd and 5th min of passive recovery in a seated position.

The fatigue index was calculated as a percentage decrement score [100 · (sprint value/best sprint value) − 100], where sprint values represented either the average workload from laboratory testing (W·kg^−1^ × 5 s^−1^) or the sprint time (s) measured on ice. The maximum percentage decrement was calculated for laboratory testing as

$$\frac{\left(\mathrm{lowest}\;\mathrm{workload}-\;\mathrm{highest}\;\mathrm{workload}\right)}{\mathrm{highest}\;\mathrm{workload}}\times 100$$

and for on-ice testing as

$$\frac{\left(\mathrm{slowest}\;\mathrm{sprint}\;-\;\mathrm{fastest}\;\mathrm{sprint}\right)}{\mathrm{fastest}\;\mathrm{sprint}}\times 100.$$

Although this results in an asymmetric formulation, the choice of denominators reflects the different physiological and mechanical characteristics of the two protocols. The laboratory assessment is based on power output, where the highest workload represents the true maximal capacity and therefore serves as the most meaningful reference point. In contrast, on-ice performance is evaluated via sprint times, where the fastest (i.e., lowest) time best reflects maximal skating speed and thus provides the appropriate benchmark. This adaptation of the standard RSA methodology described by Glaister et al. [20] was made to ensure that the fatigue metrics accurately captured performance decline within the specific constraints and measurement properties of each testing environment. The fatigue analyses were used especially in analyses of asymmetric formulas for laboratory and on-ice testing. The study was conducted in accordance with the Declaration of Helsinki and approved by the Ethics Committee of the Faculty of Physical Education and Sport, Charles University, on 25. 9. 2009 (no.: 332/2029). As part of the briefing on the research process, all participants signed an informed consent form agreeing to participate in the research.

### 2.2. Statistical Methods

The statistical analysis was carried out by using the JMP 10.0.1 statistical package (SAS Institute, Cary, NC, USA). The results are given as mean values ± standard deviations (SDs). Data were analysed using Pearson’s or Spearman’s correlation tests, selected based on the distribution of the variables to ensure appropriate validity and reliability of the results. Correlation coefficients (r) were used to detect the association between the independent and dependent variables. Values close to 0 indicate a weak association, ±0.3 indicates a moderate association, and values above ±0.7 indicate a strong association. A paired t-test was used to compare pre-to-post exercise changes in the monitored indicators between the on-ice sprint test and the laboratory protocol. The paired observations represent measurements obtained from the same participants under both testing conditions.

In accordance with the standards used for this type of research, for all statistical tests, an alpha level of *p* ≤ 0.05 was operationally defined as statistical significance.

## 3. Results

Laboratory testing was performed in 64 elite hockey players. Table 1 shows the main anthropometric, laboratory, echocardiographic and static spirometry values of the participants. We did not detect any significant pathological findings. All participants completed the entire study protocol. The results of cardiopulmonary exercise testing and repeated sprint testing are shown in Table 2.

### 3.1. Laboratory Testing Results

The VO_2_max values did not correlate with values obtained during the first bout but correlated with average workloads during the second (r = 0.26, *p* = 0.02), third (r = 0.48, *p* < 0.001), fourth (r = 0.57, *p* < 0.001) and fifth bouts (r = 0.60, *p* < 0.001). There was a trend of increasing correlation coefficient with increasing number of repetitions. VO_2_max explained only 5% of the variability of the first bout but 35% of the variability of the last bout’s average workload.

There was a correlation between VO_2_max and the sum of workloads (r = 0.49; *p* < 0.001) and both fatigue indices—the percentage workload decrement index (r = 0.44, *p* < 0.001) and percentage maximum average workload decrement (%) (r = 0.38, *p* = 0.002). Only lactate levels measured after 10 min of recovery correlated with VO_2_max (r = 0.31, *p* = 0.01).

### 3.2. On-Ice Testing Results

Thirty-two hockey players participated in the on-ice testing. For the purpose of comparison with the laboratory tests, four netminders were excluded from the sample group, as their equipment does not allow the same performance conditions on the ice as that of outfield players. The relevant data are presented in Table 3. VO_2_max showed a weak correlation with overall on-ice performance (r = 0.21, *p* = 0.24). Similarly, no significant correlation was found between VO_2_max and the on-ice fatigue index (r = 0.18, *p* = 0.31). No significant relationships were observed between VO_2_max and post-exercise blood lactate concentrations measured at 3 min (r = 0.12, *p* = 0.48) and 5 min of active recovery (r = 0.15, *p* = 0.42). These findings suggest that aerobic capacity assessed under laboratory conditions is not significantly associated with athletic sprint performance or metabolic responses on the ice.

### 3.3. Comparisons Between Laboratory and On-Ice Testing Results

No consistent or practically meaningful relationships across both age groups were observed between the results of the on-ice performance tests and the RSA laboratory tests. In the overall sample, total on-ice sprint time demonstrated a moderate association with laboratory mean power output (r = 0.35, *p* = 0.047), based on paired observations obtained from the same participants, with faster on-ice performance associated with higher relative power production in a laboratory environment. The relationship between on-ice and laboratory fatigue indices was of comparable magnitude but did not reach statistical significance (r = 0.34, *p* = 0.059). Similarly, lactate concentrations measured during recovery in the laboratory and on ice were not significantly correlated (r = 0.16, *p* = 0.388).

When the two tests were compared, mean blood lactate concentrations were significantly higher in on-ice testing compared to the laboratory at both time points: by 3.45 ± 2.40 mmol/L at the 3rd min (*p* < 0.001) and by 2.50 ± 1.80 mmol/L at the 5th min of recovery (*p* < 0.001).

In addition, the athletes exhibited greater reductions in fatigue indices during laboratory testing compared to on-ice testing. This was reflected in higher percentage decrements in performance (95.7 ± 4.8%, *p* < 0.001) and maximum performance (92.4 ± 8.5%, *p* < 0.001).

### 3.4. Comparisons Between Juniors and Seniors

Comparisons of junior and senior ice hockey players revealed significant differences in selected anthropometric and physiological characteristics. Junior players had lower body mass than senior players (*p* < 0.05), and significant differences were observed in their post-exercise lactate responses (*p* < 0.05). No statistically significant differences were observed between junior and senior ice hockey players in terms of haematological variables, cardiopulmonary exercise test outcomes, or static spirometry parameters.

To minimize the potential confounding effect associated with the combined analysis of junior and senior ice hockey players, correlation analyses between laboratory test variables and on-ice performance measures were conducted separately for each group. In junior players, a moderate correlation was identified between the maximal oxygen uptake relative to body mass (VO_2_max/kg) and total sprint time (r = 0.561, *p* = 0.046). A comparable trend was observed in senior players; however, this relationship did not reach statistical significance (r = 0.412, *p* = 0.057).

No significant associations were detected between on-ice performance and indicators of body composition or metabolic response. Specifically, body mass index was not significantly related to sprint performance in either junior (r = 0.236, *p* = 0.439) or senior players (r = 0.106, *p* = 0.648). Similarly, the blood lactate concentration measured at 3 min during low-intensity recovery following a sprint protocol showed no significant correlation with total sprint time in juniors (r = 0.145, *p* = 0.637) or seniors (r = 0.102, *p* = 0.651). The same pattern was evident for blood lactate measured at 5 min during low-intensity recovery, with no significant relationship observed in juniors (r = 0.124, *p* = 0.686) or seniors (r = 0.069, *p* = 0.760).

## 4. Discussion

Our study examined laboratory and on-ice testing in elite hockey players during the final summer training for the autumn part of the league season. The results showed that aerobic capacity, reflected by VO_2_max, was associated with performance in simulated repeated sprints. Aerobic capacity played an increasing role across repetitions during the modified anaerobic cycling test. While VO_2_max explained only 5% of workload variation in the first bout, it explained 35% in the last bout. VO_2_max was also correlated with recovery parameters predicting lactate levels at the 10th min of recovery. However, the shared variance between VO_2_max and recovery lactate was only about 10%. Although our data seemingly support the role of VO_2_max evaluation, the significance of the association between the laboratory RSA and VO_2_max is clearly insufficient for the assessment of an individual ice hockey player’s anaerobic performance. As a standalone indicator, VO_2_max has limited predictive value for physiological predispositions in ice hockey. This is further supported by the observed lack of relationship between on-ice repeated sprint testing results and VO_2_max.

The low correlation may be due, for example, to different movement patterns used in testing (cycling vs. ice skating), as well as the involvement of different muscles, skill dependence, and pacing strategies. This finding agrees with previous reports [5,6]. However, these findings were recently put to the test by Peterson et al. [9], who showed opposing results. It should be noted that the on-ice repeated shifts used in their study were substantially longer (about 23 s) than the repeated sprints used in our study. These longer shifts may be more dependent on aerobic fitness. In addition, the participants performed up to eight maximal skating repetitions. Another possible explanation for the different relationships between on-ice and laboratory testing is the method used to measure VO_2_max. Cycling on an ergometer does not reflect the typical physical effort of skating. VO_2max_ measured on ice has been shown to be higher than that in laboratory settings, with no correlation between the two methods [13].

Our laboratory testing results indicated that the relationship between aerobic fitness and simulated sprint results strengthened with increasing number of sprint repetitions. This hypothesis is supported by the close relationship observed between a “30-15 intermittent on-ice test” and VO_2_max in a study by Buchheit and co-workers using 30 s shuttle skating interspersed with 15 s passive recovery periods [21]. The positive results reported by Peterson et al. [9] were based on a skating treadmill and prolonged on-ice efforts. However, even this approach showed only a relatively weak relationship between VO_2_max and on-ice measurements. Many on-ice protocols have been proposed to test the ability of ice hockey players in their natural environment [6,9,21]. We used a modified anaerobic strength testing protocol of short, repeated sprints in the laboratory setting and on ice [17]. Although both tests comprised similar durations of effort and recovery, the relationship between the two was only weak and nonsignificant. The absence of correlation between on-ice and laboratory testing may have been due to the different magnitude and complexity of the task [22]. The validity of RSA testing protocols requires careful consideration. These protocols differ substantially in their design, duration, and recovery intervals, which affects their reliability and validity. In ice hockey, repeated sprint ability is influenced by factors such as skating technique, changes in direction, and game-specific fatigue patterns. Laboratory RSA tests may reduce variability between athletes but often fail to replicate the physiological and biomechanical demands of ice hockey.

Both tests represented predominantly anaerobic effort, as indicated by post-exercise blood lactate concentrations. However, on-ice testing was associated with significantly higher blood lactate concentrations (10.0 ± 1.5 and 10.1 ± 1.4 mmol/L at the 3rd and 5th min) compared to the ergometer (6.7 ± 1.9 and 8.1 ± 1.5 mmol/L) and therefore appeared markedly more intense than efforts performed on the ergometer.

In contrast, the performance decrement over repeated sprints was significantly lower on ice than in the laboratory. This difference is hypothesized to reflect the involvement of different muscle groups with distinct strength, and metabolic characteristics shaped by prior training. This observation agrees with findings by Durocher et al. [13], who reported higher heart rate, lactate thresholds, and VO_2_max during on-ice testing compared to laboratory conditions, with no correlation. Another interesting observation of our study arises from lactate measurements performed during active recovery. After 5 min of rest none of the hockey players showed lactate levels below 6 mmol/L and 50% of them had levels above 10 mmol/L. This suggests that during a typical match, players are unable to fully recover between shifts. This observation should be further interpreted in relation to differences between blood lactate kinetics during active and passive recovery, with faster lactate clearance typically observed during active recovery [23].

This observation agrees with intra-game measurements reported in hockey [19], rugby [19], and boxing [24]. It also indicates that repeated shifts during a game differ substantially from testing conditions, where tests usually start after a brief or fully aerobic warm-up.

The aim of this study was to analyse the performance characteristics of elite ice hockey players, including junior and senior athletes. Subgroup analyses by age category were conducted to provide additional insight into potential age-related differences in performance; however, within the overall scope of the study, these analyses are considered supplementary. Overall, both the direction and magnitude of the observed relationships were consistent across the two age groups. These findings indicate that the absence of strong correlations in the combined sample is unlikely to be attributable to underlying structural or physiological differences between junior and senior players.

There were several limitations to our study. First, voluntary participation in on-ice testing introduced potential selection bias; the on-ice test was completed by only 28 field players, which may have reduced the statistical power and affected the observed correlations. Additionally, the 60 s recovery period following the RSA test did not allow full recovery; consequently, the results of the subsequent cardiopulmonary exercise test may have been influenced by residual fatigue, which could have contributed to the lack of significant correlations between laboratory outcomes and on-ice performance. The 2–9-day interval between laboratory and on-ice testing may have affected comparability. The short time between RSA testing and VO_2_max testing may have led to underestimation of the VO_2_max results due to residual fatigue.

While the laboratory testing was performed within ideal conditions, the on-ice evaluation may have suffered due to differences in individual warm-ups and uneven equipment and motivation among the players [4,19,25]. We should also admit the potential negative role of the reduced number of players who gave their consent for the on-ice testing, decreasing the statistical power of the study. However, the main limitation may be related to the length of on-ice skating shifts chosen, including only straight-line movement. Based on recently published data, longer and more complex skating intervals may be more dependent on aerobic exercise fitness [3,9,21]. Future studies should target the game-specific validity vs. internal validity trade-off, pacing strategies and motivation differences, and neuromuscular specificity (especially skating technique).

## 5. Conclusions

The results from our laboratory and on-ice testing data confirm the role of aerobic fitness as assessed by VO_2_max capacity for the prediction of repeated anaerobic sprint ability and recovery. However, the shared variation of the different parameters with VO_2_max did not exceed 35%, making the individual prediction of subjects’ anaerobic performance from VO_2_max unreliable. Since we did not find any correlation between RSA and lactate levels during ice testing or VO_2max_ values, its role appears limited in this specific testing context as a key parameter for assessing the fitness of ice hockey players in relation to the tests we conducted. VO_2_max contributes to RSA, particularly in later bouts, but should not be used as a standalone predictor.

Adult ice hockey players demonstrated superior overall performance and post-exercise recovery capacity; however, the magnitude and direction of the relationships between VO_2_max, repeated sprint ability, and metabolic responses were comparable across both junior and adult players. It seems that on-ice testing would bring more relevant data for a more accurate assessment and training modifications. To obtain optimal results, the relationship between different tests and long-term performance should be evaluated.

## Figures and Tables

**Table 1:** Anthropometric, laboratory, echocardiographic and static spirometry characteristics of the total cohort (*n* = 64), elite juniors (*n* = 19), and senior men (*n* = 45).

Variable	Total Cohort Mean ± SD (Range)	Juniors Mean ± SD (Range)	Seniors Mean ± SD (Range)
Age (years)	21.8 ± 4.9 (17–35)	17.8 ± 0.7 (17–19)	23.9 ± 4.9 (18–35)
Anthropometric data			
Weight (kg)	84.9 ± 8.0 (67.9–100.5)	81.1 ± 7.5 (67.9–96.4)	86.9 ± 7.6 (71.6–100.5)
Height (cm)	183.5 ± 5.4 (173–195)	182.0 ± 4.8 (173–195)	184.2 ± 5.6 (175–194)
Body surface area (m^2^)	2.07 ± 0.12 (1.86–2.31)	2.02 ± 0.11 (1.86–2.29)	2.10 ± 0.11 (1.89–2.31)
Body fat (% of total body weight)	11.8 ± 3.9 (4.3–23.9)	10.4 ± 3.4 (4.3–15.6)	12.5 ± 4.0 (5.5–23.9)
Active muscle mass (% of total body weight)	50.9 ± 2.4 (42.9–55.1)	51.6 ± 2.1 (48.1–55.1)	50.5 ± 2.5 (42.9–55.0)
Blood count			
Haemoglobin (g/L)	155.7 ± 8.0 (136–177)	158.1 ± 8.6 (143–177)	154.5 ± 7.5 (136–172)
Haematocrit (%)	45.0 ± 2.1 (39–51)	45.8 ± 2.2 (43–51)	44.6 ± 2.1 (39–49)
Echocardiography			
LV mass index (g/m^2^)	86.8 ± 17.0 (46.5–138.3)	84.7 ± 22.0 (46.4–138.3)	87.9 ± 13.9 (53.5–115.9)
Relative wall thickness	0.33 ± 0.04 (0.23–0.45)	0.33 ± 0.06 (0.24–0.44)	0.34 ± 0.05 (0.23–0.45)
LV EF (%)	65.6 ± 3.9 (55–70)	65.1 ± 3.8 (57–70)	65.9 ± 3.9 (55–70)
TAPSE (mm)	26.9 ± 3.5 (19–34)	26.7 ± 3.6 (21–32)	27.0 ± 3.5 (19–34)
Static spirometry			
FVC (L/min)	6.1 ± 0.7 (5.1–8.3)	5.96 ± 0.62 (5.10–7.09)	6.25 ± 0.74 (5.12–8.30)
FVC (% of predicted)	108.5 ± 9.7 (90–133)	104.8 ± 8.2 (92–124)	110.5 ± 10.0 (90–133)

LV—left ventricle; LV EF—left ventricle ejection fraction; TAPSE—tricuspid annular plane systolic excursion; FVC—forced vital capacity.

**Table 2:** Laboratory cardiopulmonary exercise stress testing and repeated sprint ability testing results of the total cohort (*n* = 64), elite juniors (*n* = 19) and senior men (*n* = 45).

Variable	Total Cohort Mean ± SD (Range)	Juniors Mean ± SD (Range)	Seniors Mean ± SD (Range)
Cardiopulmonary exercise stress test			
VO_2_max (mL/kg/min)	50.6 ± 4.4 (40.0–61.0)	51.5 ± 4.8 (41.8–61.0)	50.1 ± 4.1 (40.0–57.5)
Maximal workload (W/kg)	4.9 ± 0.4 (4.0–5.8)	4.85 ± 0.48 (4.0–5.8)	4.89 ± 0.37 (4.2–5.6)
W170 (W/kg)	3.5 ± 0.7 (1.5–4.9)	3.50 ± 0.71 (1.5–4.7)	3.52 ± 0.70 (1.5–4.9)
Heart rate max (bpm)	184.8 ± 7.9 (169–214)	185.8 ± 9.6 (170–214)	184.2 ± 6.9 (169–206)
Anaerobic threshold (bpm)	164.0 ± 9.4 (149–190)	164.5 ± 11.7 (149–190)	163.8 ± 8.1 (149–182)
Repeated sprint ability			
Maximum lactate (mmol/L)	9.3 ± 1.7 (4.9–13.0)	8.6 ± 1.8 (4.9–11.8)	9.6 ± 1.6 (7.0–13.0)
Average workload 1st bout (W/kg/5 s)	15.3 ± 1.3 (12.0–18.3)	14.72 ± 1.11 (12.00–17.16)	15.63 ± 1.24 (13.44–18.28)
Average workload 5th bout (W/kg/5 s)	12.5 ± 1.2 (8.0–15.5)	12.94 ± 1.03 (11.23–15.47)	12.34 ± 1.24 (8.00–14.85)
% Workload decrement index (%)	−18.6 ± 7.2 (−4.0 to −40.5)	−14.1 ± 7.1 (−0.4 to −24.0)	−21.2 ± 7.6 (−9.1 to −48.5)
% Maximum average workload decrement (%)	69.4 ± 4.8 (53.2–80.4)	68.8 ± 4.4 (60.2–80.4)	69.7 ± 5.1 (53.2–80.1)
Sum of workloads (W/kg/5 × 5 s)	69.4 ± 4.8 (53.2–80.4)	68.8 ± 4.4 (60.2–80.4)	69.7 ± 5.1 (53.2–80.1)
Lactate levels during recovery (mmol/L)			
Lactate level 3rd min (mmol/L)	6.7 ± 1.9 (3.2–13.3)	6.61 ± 1.56 (4.1–9.8)	6.70 ± 2.04 (3.2–13.3)
Lactate level 5th min (mmol/L)	8.1 ± 1.5 (5.0–11.7)	7.28 ± 1.21 (5.0–10.6)	8.57 ± 1.51 (5.0–11.7)
Lactate level 10th min (mmol/L)	7.2 ± 1.6 (3.7–11.7)	6.21 ± 1.06 (4.0–8.6)	7.74 ± 1.53 (3.7–11.7)

W170—workload at HR 170 bpm.

**Table 3:** On-ice testing: total cohort (*n* = 29), elite juniors (*n* = 13) and senior men (*n* = 16).

Variable	Total Cohort Mean ± SD (Range)	Juniors Mean ± SD (Range)	Seniors Mean ± SD (Range)
Repeated sprint ability			
Sprint time 1st bout (s)	5.00 ± 0.28 (4.43–5.51)	4.87 ± 0.32 (4.43–5.62)	5.29 ± 0.43 (4.70–6.48)
Sprint time 5th bout (s)	5.22 ± 0.20 (4.82–5.64)	5.24 ± 0.34 (4.88–6.20)	5.40 ± 0.39 (4.82–6.33)
% Sprint time increment index (%)	4.1 ± 1.6 (1.3–7.8)	7.6 ± 3.0 (1.8–13.0)	2.4 ± 5.2 (6.0–12.5)
% Maximum sprint time increment (%)	8.3 ± 2.9 (3.9–14.9)	9.4 ± 2.6 (6.1–14.9)	7.9 ± 2.8 (4.0–14.0)
Sum of sprint times (s)	25.52 ± 0.81 (24.03–26.95)	25.49 ± 1.59 (24.03–30.03)	26.59 ± 1.96 (24.82–31.85)
Lactate levels during recovery (mmol/L)			
Lactate level 3rd min (mmol/L)	10.0 ± 1.5 (6.4–12.7)	9.72 ± 1.69 (6.4–12.7)	10.16 ± 1.28 (7.2–12.6)
Lactate level 5th min (mmol/L)	10.1 ± 1.4 (6.7–12.8)	9.88 ± 1.26 (7.1–11.1)	10.04 ± 1.48 (6.7–12.8)

## Data Availability

Data can be obtained from the corresponding author upon reasonable request.

## Abbreviations

The following abbreviations are used in this manuscript:

ECG	electrocardiogram
RSA	repeated sprint ability
VO_2_max	maximum oxygen uptake
WT	Wingate Anaerobic Test
